# Dual Modulation of Adipogenesis and Apoptosis by PPARG Agonist Rosiglitazone and Antagonist Betulinic Acid in 3T3-L1 Cells

**DOI:** 10.3390/biomedicines13061340

**Published:** 2025-05-30

**Authors:** Patsawee Sriboonaied, Pornwipa Phuangbubpha, Puretat Saetan, Purin Charoensuksai, Adisri Charoenpanich

**Affiliations:** 1 Department of Biology, Faculty of Science, Silpakorn University, Nakhon Pathom 73000, Thailand; sriboonaied_p@su.ac.th (P.S.); phuangbubpha_p@su.ac.th (P.P.); saetan_p2@silpakorn.edu (P.S.); 2 Department of Biomedicine and Health Informatics and Bioactives from Natural Resources Research Collaboration for Excellence in Pharmaceutical Sciences (BNEP), Faculty of Pharmacy, Silpakorn University, Nakhon Pathom 73000, Thailand; charoensuksai_p@su.ac.th

**Keywords:** 3T3-L1, apoptosis, adipogenesis, PPARG modulators, cell viability assays

## Abstract

**Background/Objectives:** Disruptions in adipose tissue dynamics contribute to obesity-related metabolic disorders, emphasizing the need for targeted therapies focusing on adipose tissue cells, including progenitor cells and adipocytes. Peroxisome proliferator-activated receptor gamma (PPARG) ligands are potent insulin sensitizers used in type 2 diabetes treatment. This study investigated the effects of rosiglitazone, a PPARG agonist, and betulinic acid, a PPARG antagonist, on adipogenesis and apoptosis in 3T3-L1 pre-adipocytes. **Method:** 3T3-L1 pre-adipocytes were treated with rosiglitazone or betulinic acid during adipogenic differentiation. Lipid droplet formation was used to evaluate adipogenesis. Cell growth and cell death were assessed using the resazurin-based cell viability assay, trypan blue exclusion assay, LDH assay, and Annexin V/PI staining. Quantitative PCR was conducted to examine the expression of genes associated with adipogenesis and apoptosis. **Results:** Betulinic acid reduced adipogenesis only when administered daily for eight days. Rosiglitazone did not alter the overall lipid quantity; however, it promoted a shift toward fewer but larger lipid droplets. Both compounds increased *Adipoq* and *Cfd* expression, and betulinic acid also elevated *Fabp4*. Rosiglitazone induced stronger cell aggregation. Despite increased cell death, overall viability was maintained. Apoptotic cell death was enhanced by both compounds and confirmed via Annexin V/PI staining and flow cytometry, accompanied by downregulation of *Ccnd1* and *Bcl2*. Additionally, rosiglitazone markedly increased the expression of *Cebpa*, a key regulator that can modulate lipid droplet formation and the balance between cell growth and death. **Conclusions:** Rosiglitazone and betulinic acid differentially modulate adipogenesis and apoptosis in 3T3-L1 cells, revealing a complex interplay between lipid accumulation and programmed cell death. Together, the findings underscore the potential of dual PPARG-targeting approaches for metabolic disease interventions.

## 1. Introduction

The critical role of adipose tissue in metabolic regulation and its connection to metabolic syndromes, such as diabetes and cardiovascular diseases, stems from its dual function as both an energy reserve and an endocrine organ. This dual functionality is pivotal, as adipose tissue not only stores energy but also secretes various regulatory adipokines and cytokines [1]. The increasing prevalence of obesity has profound implications for human health, primarily because of dysregulated adipose tissue homeostasis characterized by uncontrolled cell proliferation, differentiation, and apoptosis among adipogenic progenitors and mature adipocytes [2,3]. This imbalance leads to adipose tissue dysfunction, highlighting the necessity for therapeutic interventions targeting adipose tissue metabolic activity [4].

Strategies proposed to mitigate adipose tissue dysfunction encompass efforts to modulate the fate of adipose cells, including reducing adipogenic differentiation in mesenchymal stem cells or adipogenic progenitor cells, enhancing lipolysis, decreasing lipogenesis, and promoting thermogenesis [3,4,5]. Additionally, a synergistic approach that combines reducing adipogenesis with encouraging apoptosis has been suggested as a potential therapeutic pathway [5,6]. The process of adipogenic differentiation is defined by a series of stages: initial growth arrest, mitotic clonal expansion (MCE) of committed cells, changes in gene expression, and terminal differentiation [7]. Central to this process is the role of peroxisome proliferator-activated receptor gamma (PPARG), a master regulator that orchestrates the transcriptional machinery governing adipogenesis. PPARG has been recognized as the master regulator of adipose tissue biology, playing a critical role in adipocyte differentiation, maintenance, and function [8].

The management of type II diabetes using Thiazolidinediones (TZDs), which are PPARG agonists, is controversial because of their known adverse effects, including increased body fat, bone loss, edema, liver dysfunction, and potential cardiovascular risks [9,10,11]. These side effects have prompted the search for alternative PPARG ligands that can enhance insulin sensitivity without promoting excessive adipogenesis [12,13]. Rosiglitazone, a TZD well-known for its role in promoting insulin sensitivity and regulating lipid metabolism with a relatively low risk of gastrointestinal side effects, was chosen as the representative PPARG agonist in this study [14,15,16]. Studies have shown that moderate inhibition of PPARG activity can prevent insulin resistance and obesity induced by high-fat diets, while PPARG antagonists can improve insulin sensitivity and promote the browning of white adipose tissue, potentially offering a safer therapeutic approach [17,18]. In this context, betulinic acid has emerged as a promising natural PPARG antagonist [19]. Similar to GW9662, a potent synthetic PPARG antagonist, betulinic acid, a natural pentacyclic triterpenoid derived from birch bark, has been shown to ameliorate metabolic dysfunction associated with steatotic diseases [20,21]. Unlike GW9662, however, BA offers additional benefits because of its natural origin, potentially lower toxicity, and broader metabolic effects. These include the inhibition of adipogenesis, enhancement of glucose uptake, promotion of brown adipocyte differentiation, and stimulation of osteogenesis, making it a compelling candidate for metabolic disease therapy [22,23]. Interestingly, while both betulinic acid and GW9662 are PPARG antagonists, betulinic acid has demonstrated potent anti-inflammatory activity [24,25] and has been shown to attenuate oxidative stress, reduce renal damage, and protect against ischemia/reperfusion-induced renal injury [26,27,28]. In contrast, GW9662 has been reported to reverse the protective effects of lipopolysaccharide (LPS) in renal ischemia-reperfusion, potentially exacerbating renal injury under similar conditions [29]. This difference highlights the broader therapeutic potential of betulinic acid as a PPARG antagonist, offering a more favorable safety profile with multi-targeted benefits, including anti-inflammatory, antioxidant, and tissue-protective effects, making it a superior choice for studies aiming to explore safer therapeutic strategies for metabolic and inflammatory diseases.

To address these complex interactions, this study aims to investigate the differential effects of rosiglitazone and betulinic acid on adipogenesis and apoptosis in 3T3-L1 cells, providing novel insights into the potential therapeutic roles of these PPARG modulators in metabolic regulation. This study investigated the mechanistic effects of betulinic acid and rosiglitazone on 3T3-L1 cells, a widely used in vitro model of adipogenesis, across distinct stages of differentiation. Morphological changes, lipid droplet formation, and lipid accumulation were assessed following administration of the PPARG modulators at specific phases of adipogenesis: early, middle, late, and throughout the entire differentiation period. Cell proliferation dynamics were evaluated by analyzing cell aggregation patterns, viability, and death. Apoptotic cell death was further characterized using Annexin V/PI staining combined with flow cytometry, as well as gene expression analysis. Importantly, this study provides novel insights into the presence of cell aggregates during 3T3-L1 adipogenesis, revealing that these regions harbor significant populations of apoptotic cells, a process further exacerbated by both rosiglitazone and betulinic acid. *Ccnd1* and *Bcl2* were identified as potential key regulators of this response. This study provides preliminary evidence that may contribute to a better understanding of adipose tissue dynamics and the complex interplay between differentiation and cell death in adipocyte development.

## 2. Materials and Methods

### 2.1. Cell Culture

Mouse pre-adipocyte 3T3-L1 cells (JCRB9014) were obtained from the Japanese Collection of Research Bioresources Cell Bank (JCRB, Osaka, Japan). Cells were maintained in tissue culture flasks (T-25, T-75) (Nunclon, Shanghai, China) with complete growth medium (CGM) containing Dulbecco’s Modified Eagle Medium with low glucose (Hyclone, Logan, UT, USA), supplemented with 10% fetal bovine serum (Hyclone), 2 mM L-glutamine (Corning Inc., Corning, NY, USA), and 1% penicillin/streptomycin (Hyclone) at 37 °C with 5% CO_2_.

### 2.2. Adipogenic Differentiation

**Figure 1:**
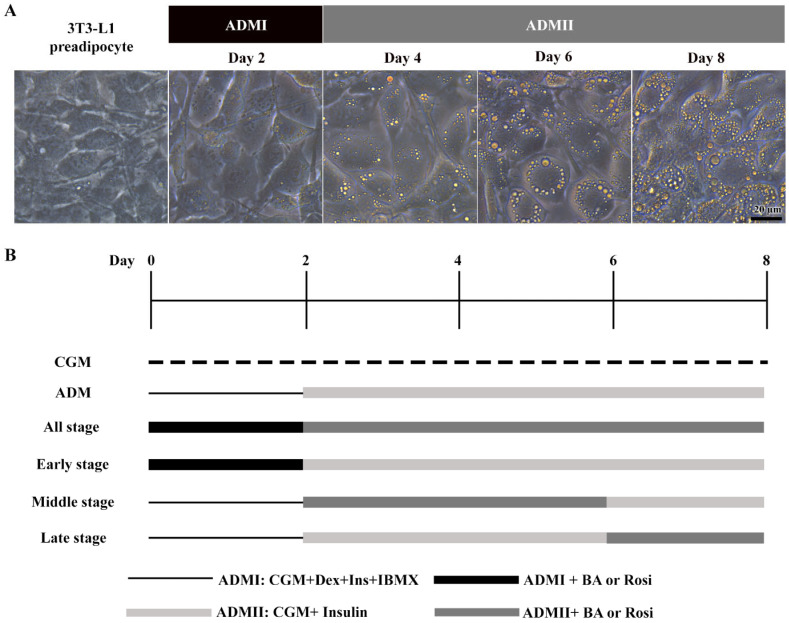
Stages of adipogenic differentiation in 3T3-L1 cells. (**A**) Morphological changes observed in 3T3-L1 cells, transitioning from spindle-shaped to enlarged, round cells accompanied by the accumulation of lipid droplets. (**B**) Schematic representation of the adipogenic induction process and the various stages of betulinic acid (BA) and rosiglitazone (rosi) treatment.

The 3T3-L1 cells underwent adipogenic differentiation treated with adipogenic differentiation media (ADM). Initially, cells received ADMI, consisting of CGM with 1 µM dexamethasone (Sigma, St. Louis, MO, USA), 10 µg/mL h-insulin (Sigma), and 500 µM isobutylmethylxanthine (IBMX) (Sigma), for the first two days. Subsequently, the medium was switched to ADMII, a CGM supplemented with 10 µg/mL insulin, for an additional 6 days (Figure 1).

### 2.3. Cell Viability Assays

The 3T3-L1 pre-adipocytes were seeded in 96-well tissue culture plates (Nunclon^TM^ Delta Surface, Roskilde, Denmark) at a density of 5000 cells per well and cultured for 48 h before undergoing adipogenic induction combined with treatment. Betulinic acid was administered at concentrations of 0, 5, 10, and 20 µM for initial screening and at 20 µM for the remainder of the experiments. Rosiglitazone was used at a concentration of 2 µM. Resazurin sodium salt (Sigma) was dissolved in phosphate-buffered saline (PBS) to create a 44 µM stock solution, which was then diluted with CGM to achieve a final concentration of 4.4 µM. The cells were incubated in the dark for 4 h before absorbance was measured at 570 nm and 600 nm using a microplate reader (PACKARD, A153601, Palo Alto, CA, USA).

### 2.4. Oil Red O Staining

Oil Red O staining was utilized to examine intracellular lipid accumulation. The 3T3-L1 cells were washed with PBS and fixed with 4% paraformaldehyde. A 0.5% (*w*/*v*) Oil Red O solution (Sigma) was mixed with DI water at a 3:2 ratio and allowed to stand for 10 min. The working solution was then filtered and applied to the cells for 15 min at room temperature for staining. Images were captured from five different areas of each well in triplicate using an inverted microscope (Leica, DMi1, Wetzlar, Germany). The area of lipid droplets stained with Oil Red O was evaluated using ImageJ software (version 2.14.0/1.54f) (National Institutes of Health, Bethesda, MD, USA).

### 2.5. BODIPY/DAPI Staining

BODIPY/DAPI staining was performed to visualize lipid droplets alongside the nuclei for detailed analysis. After adipocyte differentiation, cells were washed with PBS and fixed with 4% paraformaldehyde for 15 min, then permeabilized with 0.1% Triton X-100 in PBS (*v/v*) for another 15 min. For the staining, cells were first stained with 10 µg/mL BODIPY 493/503 (Sigma) for 10 min and then counterstained with 1 µg/mL DAPI (Sigma) for 5 min. The lipid droplets and nuclei were visualized under an inverted fluorescence microscope (Olympus, CKX53, Tokyo, Japan) using the blue and UV channels, respectively. The areas of lipid droplets and nuclei in each condition were analyzed using ImageJ software (version 2.14.0/1.54f). The fluorescence ratio (BODIPY/DAPI) was calculated by dividing the area of lipid droplets by the area of nuclei [12].

### 2.6. Hematoxylin and Eosin Staining

The 3T3-L1 cells were fixed in 4% paraformaldehyde for 10 min at room temperature. Following fixation, cells were rinsed twice with PBS to remove residual fixative. Nuclei were stained with hematoxylin for 5 min, followed by a brief rinse with distilled water to remove excess stain. To enhance contrast and cytoplasmic visualization, cells were then counterstained with eosin for 3 min before a final rinse with distilled water.

### 2.7. Lactate Dehydrogenase Activity Assay

The assay for lactate dehydrogenase (LDH) activity in the culture media serves as an indicator of cell damage or death. This indirect method detects LDH activity by monitoring the conversion of NAD to NADH. The procedure was carried out in accordance with the protocol provided by the manufacturer (Sigma). Initially, media samples were collected on days 4 and 8, followed by centrifugation at 10,000× *g* for 5 min to remove cell debris. A total of 50 µL of the media supernatant was mixed with 50 µL of a prepared reaction master mix. The mixture was then incubated, and absorbance was measured at 450 nm at 5 min intervals for up to 60 min using a Biochrom EZ Read 2000 microplate reader (Biochrom, Cambridge, UK) with software version 1.3.0.0. After that, the ΔA450 value was determined by calculating the change in measurement from the initial time point (Tinitial) to the final time point (Tfinal), normalized with respect to the absorbance of the CGM blank.

∆A450=(A450)final−(A450)initial−CGMblank

The ΔA450 values were then converted to the amount of NADH for each sample by comparison with a standard curve. The LDH activity of a given sample was determined using the following equation:

LDH activity=(Amount of NADH(mole))/((Tfinal             −Tinitial)×Sample volume(mL))×Sample dilution factor

### 2.8. Propidium Iodide Staining

Propidium iodide (PI) staining was employed to visualize dead cells within populations of live cells, as PI can penetrate cells that have lost membrane integrity. Cells were gently washed once with PBS and then incubated with PI (Immunotools, Friesoythe, Germany) in PBS for 20 min.

### 2.9. Annexin-V and Propidium Iodide Staining

The cells were rinsed with PBS twice and stained with Annexin V/PI (Immunotools) in darkness for 20 min. The fluorescence area and intensity of Annexin V and PI were quantified using ImageJ software (version 2.14.0/1.54f). The corrected total cell fluorescence (CTCF) was determined according to the provided equation [13].

Mean of CTCF=Integrated density−(area of selected cells×mean of background)

### 2.10. Trypan Blue Assay

The 3T3-L1 cells were first trypsinized. Subsequently, trypan blue 0.04% *w*/*v* was mixed with the cell suspension at a 1:2 dilution. The numbers of live and dead cells were then quantified using a hemocytometer.

### 2.11. Flow Cytometry

Cells were collected and resuspended at a concentration of 100,000 cells in 500 µL of binding buffer containing 5 µL of Annexin V and 2 µL of PI dyes (Immunotools). The samples were incubated at room temperature for 20 min. Flow cytometry was performed using the Attune NxT (Thermo Fisher Scientific, Waltham, MA, USA), and these data were analyzed with Attune NxT software version 4.2.0 (Thermo Fisher, Waltham, MA, USA).

### 2.12. Mitochondrial Staining

The 3T3-L1 cells were incubated with 200 nM MitoNIR (Abcam, Waltham, MA, USA) in PBS for 1 h at 37 °C. Afterward, the cells were washed with PBS and counterstained with 0.1 μg/mL Hoechst (Abcam, Waltham, MA, USA) for 5 min. The fluorescence intensity was analyzed using ImageJ software (version 2.14.0/1.54f). The mean of CTCF/cell was determined by dividing the calculated mean of CTCF by the number of nuclei [30].

### 2.13. Gene Expression Analysis

The cells were treated with 20 µM of BA and 2 µM rosiglitazone in an adipogenic differentiation medium for 2 or 6 days. Following treatment, cells were harvested, and RNA extraction was performed using the total RNA mini kit (Favorgen, Ping Tung, Taiwan), following the manufacturer’s instructions. The synthesis of complementary DNA was conducted using the iScript reverse transcription supermix (Biorad, Hercules, CA, USA). Real-time PCR amplification was performed using the iTaq Universal SYBR Green Supermix (Biorad) on the Applied BiosystemsTM 7500 real-time PCR system. Cycling conditions consisted of an initial denaturation step at 95 °C for 2 min, followed by 40 cycles of denaturation at 95 °C for 15 s and annealing/extension at 60 °C for 1 min. Gene expression was normalized to 18S gene expression levels. Cycle threshold (Ct) values were determined for each sample, and relative gene expression levels were calculated using the 2^−ΔΔCt^ method, with a two-fold change cut-off being implemented. Melting curve analysis was performed to confirm amplicon specificity. The primers sequences targeting the adipogenic used were [31,32,33,34,35,36]: *Pparg2*: sense 5′-TGTCGGTTTCAGAAGTGCCTTG-3′ and antisense 5′ TTCAGCTGGTCGATATCACTGGAG-3′; *Adipoq*: sense 5′-GATGCAGGTCTTCTTGGTC CTAA-3′ and antisense 5′-GGCCCTTCAGCTCCTGTC-3′; *Fabp4*: sense 5′-CCAATGAGCAA GTGGCAAGA-3′ and antisense 5′-GATGCCAGGCTCCAGGATAG-3′; *Cfd*: sense 5′-CTGGGAGCGGCTGTATGT-3′ and antisense 5′-CACGGAAGCCATGTAGGG-3′; *Lep*: sense 5′-TCCCTGCCTCAGACCAGTG-3′ and antisense 5′-TAGAGTGAGGCTTCCAGGACG-3′; *Cebpa*: sense 5′-GAGCCGAGATAAAGCCAAACA-3′ and antisense 5′-CGGTCATTGTCAC TGGTCAACT-3′. The Cell cycle and apoptosis primer sequence used were [37,38,39,40,41,42]: *Trp53*: sense 5′-TAAAGGATGCCCATGCTACAG-3′ and antisense 5′-GACCGGGAGGATTGTG TCTC-3′; *Cdkn1a*: sense 5′-AGTACTTCCTCTGCCCTGCTG-3′ and antisense 5′-GCGCTTG GAGTGATAGAAATCTG-3′; *Cdk2*: sense 5′-CCTGGATGAAGATGGACG-3′ and antisense 5′-TCAGAGCCGAAGGTGGG-3′; *Ccnd1*: sense 5′-GTGAGGAGCAGAAGTGCGAAGA-3′ and antisense 5′-CGGCAGTCAAGGGAATGGT-3′; *Bax*: sense 5′-CCAGGATGCGTCCACC AAGA-3′ and antisense 5′-GGTGAGGACTCCAGCCACAA-3′; *Bcl2:* sense 5′-TGAGTAC CTGAACCGGCATCT-3′ and antisense 5′-GCATCCCAGCCTCCGTTAT-3′.

### 2.14. Statistical Analysis

All analyses were performed using SPSS V25 software (IBM Corp., Armonk, NY, USA). Data were expressed as the mean ± standard deviation (SD) obtained from three experimental replicates. Normality was assessed using the Shapiro–Wilk test, and homogeneity of variances was assessed using Levene’s test. A one-way analysis of variance (ANOVA) was employed to determine whether there were significant differences between group means. In the event of a significant ANOVA result, post-hoc tests, Tukey’s HSD and Dunnett C, were conducted to identify specific differences between groups. A significance level of *p* < 0.05 or *p* < 0.01 was applied.

### 2.15. Image Analysis and Software

All images were captured using Olympus cellSens standard software (version 2.3) with appropriate fluorescence filter sets. The UV channel (excitation: 340–390 nm, emission: 420 nm) was used for DAPI and Hoechst staining, the blue channel (excitation: 460–495 nm, emission: 510 nm) for Annexin V and BODIPY staining, and the green channel (excitation: 530–550 nm, emission: 575 nm) for propidium iodide and MitoTracker Red staining. Image processing and quantitative analysis were performed using ImageJ version 2.14.0/1.54f. Fluorescence intensity measurements were conducted using the “Analyze Particles” function in ImageJ software (version 2.14.0/1.54f), following background subtraction and thresholding to ensure precise signal quantification.

## 3. Results

### 3.1. Adipogenic Differentiation and Changes in Cell Morphology

When exposed to ADM, the 3T3-L1 cells exhibited a notable change in cell morphology, transitioning from a fibroblastic appearance to an enlarged round shape within two days, accompanied by the formation of visible small lipid droplets at day 4 (Figure 1A). This transformation was further characterized by a continuous increase in both the number and size of lipid droplets (Figure 1A).

**Figure 2:**
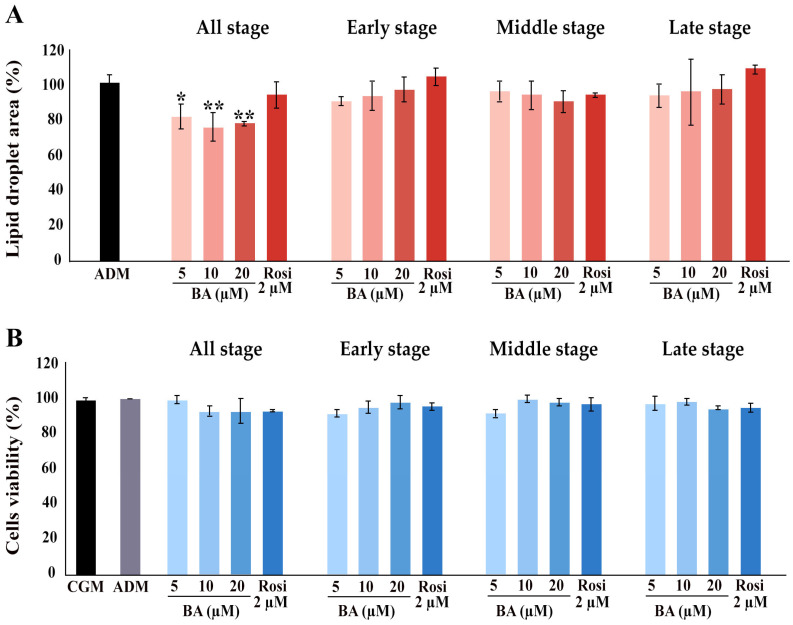
Influence of betulinic acid (BA) and rosiglitazone (Rosi) treatment on lipid droplet formation and cell viability during adipogenic differentiation of 3T3-L1 cells. (**A**) Quantification of lipid droplet area, as determined by Oil Red O staining and ImageJ (version 2.14.0/1.54f) analysis. (**B**) Assessment of 3T3-L1 cell viability with a resazurin-based assay. Statistical significance is indicated with * *p* < 0.05, ** *p* < 0.01.

Further, the implementation of betulinic acid at concentrations of 5 µM, 10 µM, and 20 µM, along with rosiglitazone at a concentration of 2 µM, was carried out in four different regimens covering all stages (8 days), the early stage (day 0–2), the middle stage (day 2–6), and the late stage (day 6–8) (Figure 1). The results revealed that treatment with betulinic acid in concentrations ranging from 5 µM to 20 µM resulted in a decrease in total lipid droplet formation, but this effect required continuous treatment over 8 days (Figure 2A). In contrast, rosiglitazone did not significantly alter the amount of lipid droplet formation (Figure 2A). Cell viability was consistently maintained at all stages, as confirmed by the resazurin cell viability assay (Figure 2B).

**Figure 3:**
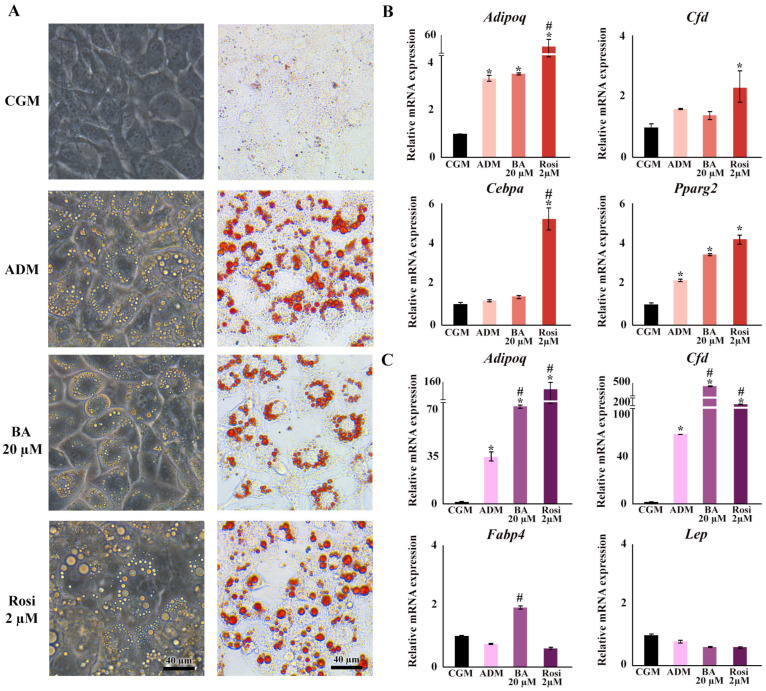
Changes in cell morphology, lipid droplet formation, and gene expression during adipogenesis following treatments with betulinic acid and rosiglitazone. (**A**) Phase-contrast microscopy showcases live-cell morphology, while Oil Red O staining highlights lipid droplets (×10 objective). (**B**,**C**) present the relative mRNA expression of adipogenic genes on days 2 and 6, respectively. Data normalized to 18S rRNA of the control group (CGM) are expressed as means ± SD. An asterisk (*) indicates *p* < 0.05 compared with CGM; a hash (#) indicates *p* < 0.05 compared with the adipocyte differentiation medium (ADM) with a 2-fold cut-off.

Regarding changes in adipocyte cell morphology, the introduction of betulinic acid resulted in an increase in the number of small lipid droplets, whereas rosiglitazone promoted the development of larger lipid droplets (Figure 3A). Gene expression analysis confirmed the upregulation of adipogenic markers by ADM, specifically adiponectin (*Adipoq*) and peroxisome proliferator-activated receptor gamma 2 (*Pparg2*), by day 2, and *Adipoq* along with small molecule-induced complement factor D/Adipsin (*Cfd*) by day 6 (Figure 3B,C). On day 2, the addition of rosiglitazone significantly boosted the expression of adipogenic markers compared with ADM alone, particularly elevating the levels of *Cebpa* and *Cfd* (Figure 3B). Moreover, rosiglitazone considerably enhanced the effects of ADM by increasing *Adipoq* expression nearly 15-fold (Figure 3B). In contrast, betulinic acid did not significantly influence the effects of ADM at day 2. Nevertheless, it increased the expression of fatty acid binding protein 4 (*Fabp4*) and further intensified the upregulation of *Adipoq* and *Cfd* by day 6 (Figure 3B,C). The delayed upregulation of *Adipoq*, *Cfd*, and *Fabp4* by betulinic acid, observed on day 6, supports the notion that continuous treatment is required to modulate key adipogenic pathways and exert a measurable effect on lipid accumulation.

### 3.2. Betulinic Acid and Rosiglitazone Enhanced Cell Aggregation While Betulinic Acid Reduced Lipid Accumulation

**Figure 4:**
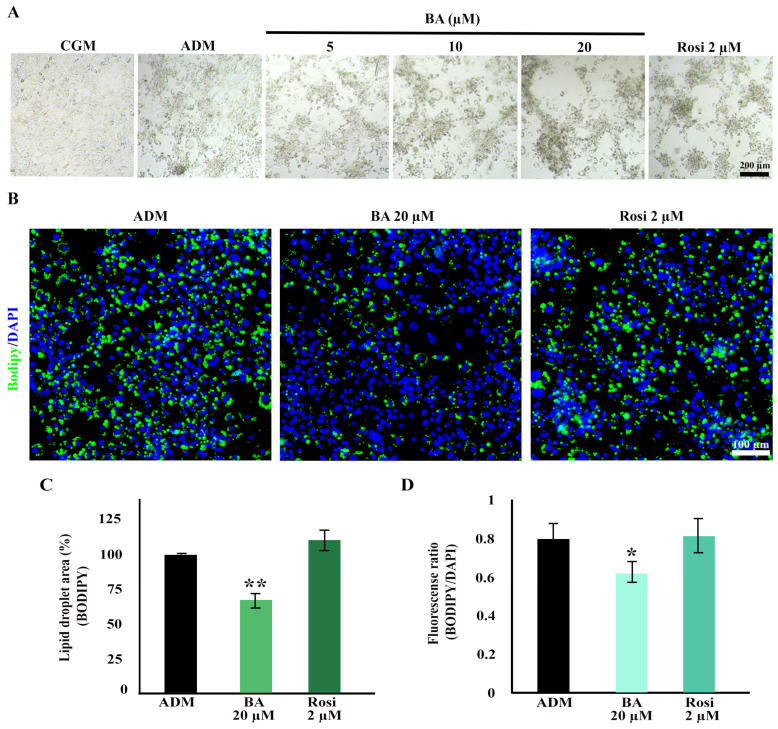
Enhanced cell aggregation during adipogenic differentiation of 3T3-L1 cells with betulinic acid and rosiglitazone. (**A**) Low-magnification bright-field microscopic images display cell aggregation patterns (×4 objective). (**B**) Bodipy/DAPI staining highlights lipid droplets in green and nuclei in blue, revealing densely aggregated cells with fewer lipid droplets in cells treated with betulinic acid (×10 objective). Quantitative analyses of the lipid droplet-stained area (**C**) and the lipid droplet-stained area normalized to the number of nuclei (**D**). Statistical significance is denoted by * *p* < 0.05, ** *p* < 0.01.

Cell aggregation resulting from mitotic clonal expansion during the adipogenesis of 3T3-L1 cells was observed at low magnification. Interestingly, both betulinic acid and rosiglitazone appeared to enhance cell aggregation further (Figure 4A). Further analysis using BODIPY/DAPI staining revealed a high cell density in the aggregated area. Notably, betulinic acid exhibited a reduction in lipid droplet formation within the aggregated area (Figure 4B).

Additionally, a decrease in total lipid droplets and lipid droplets per cell induced by betulinic acid was confirmed through BODIPY/DAPI staining. Betulinic acid decreased total lipid droplets by 24.66 ± 0.73% (Figure 4C) and, when analyzed in combination with a number of cells based on DAPI staining of nuclei, reduced lipid droplet/cells by 18.76 ± 2.01% (Figure 4D).

### 3.3. Morphological Changes and Characterization of Cell Death in 3T3-L1 Differentiation

**Figure 5:**
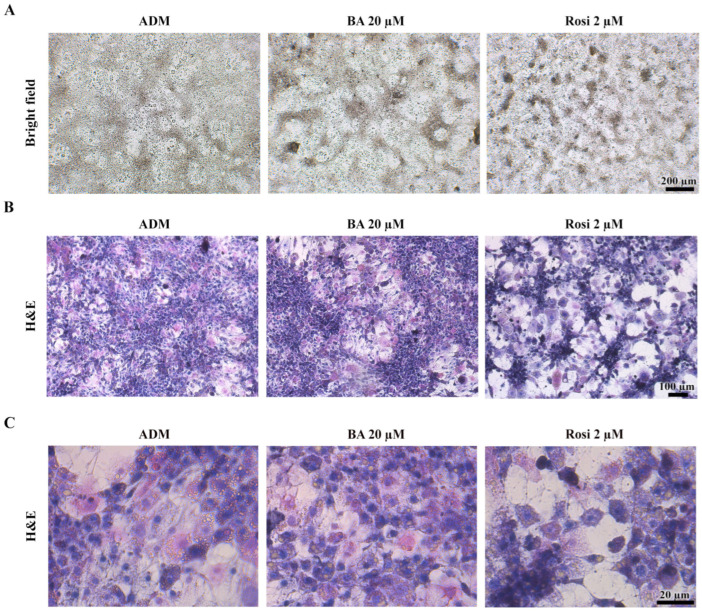
Cell aggregation patterns during adipogenic differentiation of 3T3-L1 cells supplemented with betulinic acid or rosiglitazone. (**A**) Images of live cells at day 8 (×4 objective) and corresponding hematoxylin–eosin staining show cell morphology and increased basophilic cytoplasm induced by rosiglitazone. (**B**,**C**) represent the same stained sections observed at low (×10 objective) and high (×40 objective) magnification, respectively.

Given that the resazurin cell viability assay revealed no significant alterations in cell viability (Figure 2B), our attention turned to the intriguing observation that both betulinic acid and rosiglitazone appeared to modulate the pattern of mitotic clonal expansion. Bright field observation revealed that betulinic acid increased the size of a large, dark aggregated area, while rosiglitazone induced denser aggregation, albeit in smaller and more evenly distributed clusters (Figure 5A). Hematoxylin and eosin staining revealed two distinct cell morphologies: enlarged spread cells in the lower layer and non-aggregated area and densely packed small cells in the upper layer of the aggregated area (Figure 5B). Additionally, rosiglitazone treatment induced a transition in 3T3-L1 cells from acidophilic to basophilic cytoplasm, as demonstrated by hematoxylin–eosin staining (Figure 5C).

**Figure 6:**
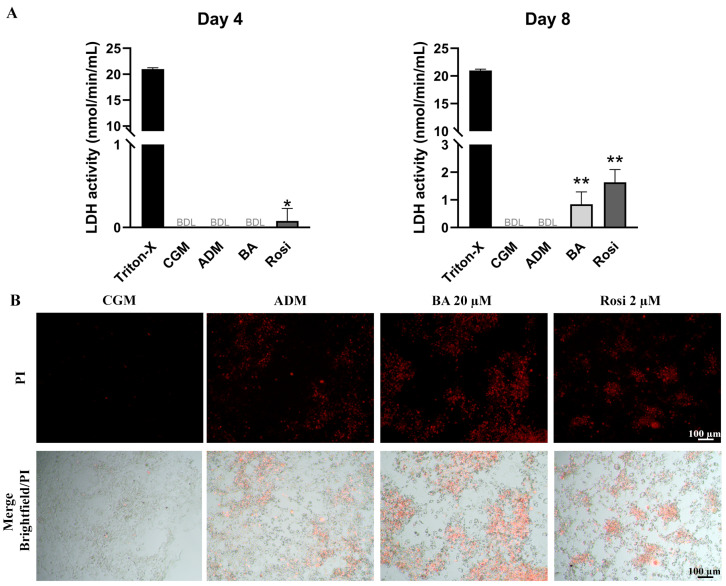
Detection of cell death in 3T3-L1 cells during adipogenesis supplemented with betulinic acid or rosiglitazone. (**A**) LDH activity was measured on days 4 and 8. Statistical significance is indicated with * *p* < 0.05. ** *p* < 0.01 (**B**) Presence of dead cells in aggregated 3T3-L1cell populations visualized with propidium iodide staining after 8 days in ADM (×4 objective).

LDH assay results on day 4 revealed that rosiglitazone treatment induced a detectable level of cell death during adipogenesis (Figure 6A). By day 8, LDH activity remained undetectable in both control groups (CGM and ADM); however, cells treated with the combination of betulinic acid and rosiglitazone showed a significant increase in LDH release, indicating an enhancement of adipogenesis-associated cell death (Figure 6A). Live staining with propidium iodide further confirmed the occurrence of adipogenesis-associated cell death (Figure 6B). These characteristics of cell aggregation and cell death were not detected in the absence of adipogenic induction (Figure S1), while some lipid droplet accumulation could be observed with rosiglitazone alone, even without the adipogenic differentiation media (Figure S1).

### 3.4. Changes in Cell Number and Mitochondrial Activity

**Figure 7:**
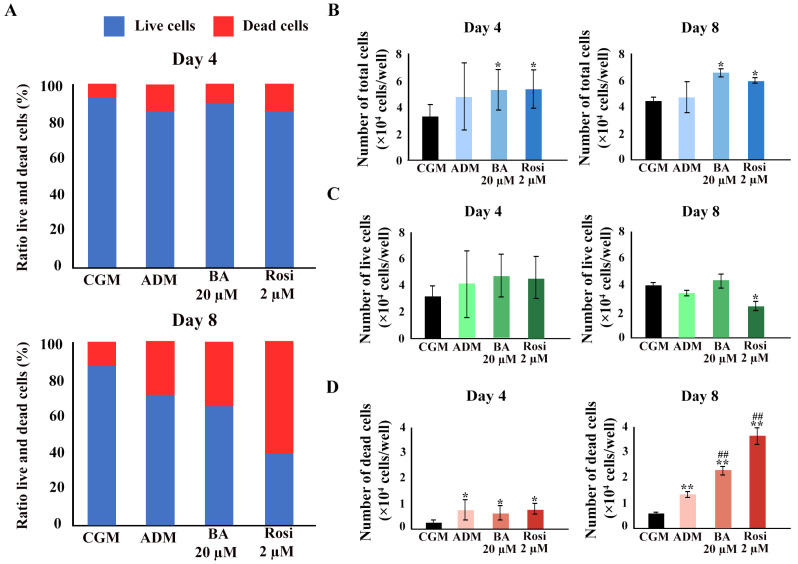
The Trypan blue exclusion assay confirmed an increase in total cell number because of betulinic acid and rosiglitazone. (**A**) The ratio of live to dead cells at days 4 and 8. Total cell numbers (**B**), number of live cells (**C**), and number of dead cells (**D**) are shown. Data are expressed as means ± SD (*n* = 3). * *p* < 0.05 and ** *p* < 0.01 compared with CGM, while ## *p* < 0.01 compared with ADM.

We hypothesized that cell viability was either maintained because of a higher number of total cells during the phase of clonal expansion in adipogenesis or that increased mitochondrial activity contributed to the unchanged cell viability detected with mitochondrial function assays such as resazurin-based assays. A Trypan blue exclusion assay was performed. An increase in the ratio of dead cells was observed (Figure 7A). Betulinic acid and rosiglitazone significantly increased the total cell numbers on days 4 and 8 (Figure 7B). While the cell numbers increased, the number of live cells did not change, except on day 8, when rosiglitazone reduced the number of viable cells (Figure 7C). The ADM increased the number of dead cells, an effect that was further enhanced by the addition of betulinic acid or rosiglitazone (Figure 7D).

**Figure 8:**
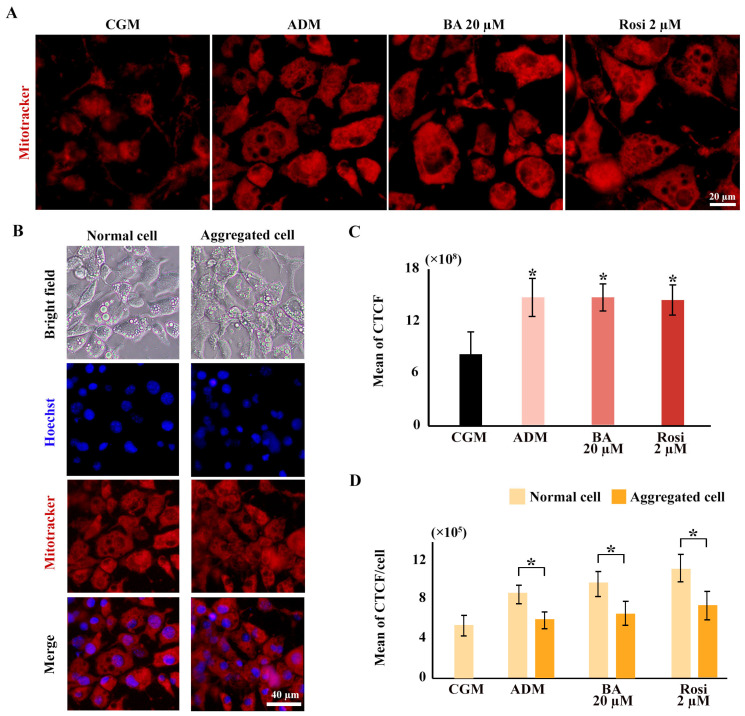
Elevated mitochondrial density during 3T3-L1 adipogenesis potentially obscures reductions in cell viability in metabolic tracking assays. (**A**) Mitochondrial staining images reveal increased mitochondrial density across all ADM conditions (×40 objective). (**B**) Mitotracker co-staining with Hoechst highlights differences in mitochondrial and cell density (×10 objective). (**C**) Corrected total cell fluorescence (CTCF) quantifies mitochondrial density, and (**D**) ratios normalized to cell numbers from Hoechst staining compare normal and aggregated areas. Statistical significance is indicated with * *p* < 0.05.

Mitochondrial staining was conducted using a mitotracker, revealing an increased distribution of mitochondria with adipogenic induction but not from the addition of betulinic acid or rosiglitazone (Figure 8A,C). The ratio of mitochondrial density to cell numbers indicated a higher mitochondrial density in normal cell areas compared with aggregated cells; however, this difference was not statistically significant with the addition of betulinic acid or rosiglitazone (Figure 8B,D).

### 3.5. Enhancement of Apoptotic Cell Death During Adipogenesis by Betulinic Acid and Rosiglitazone

**Figure 9:**
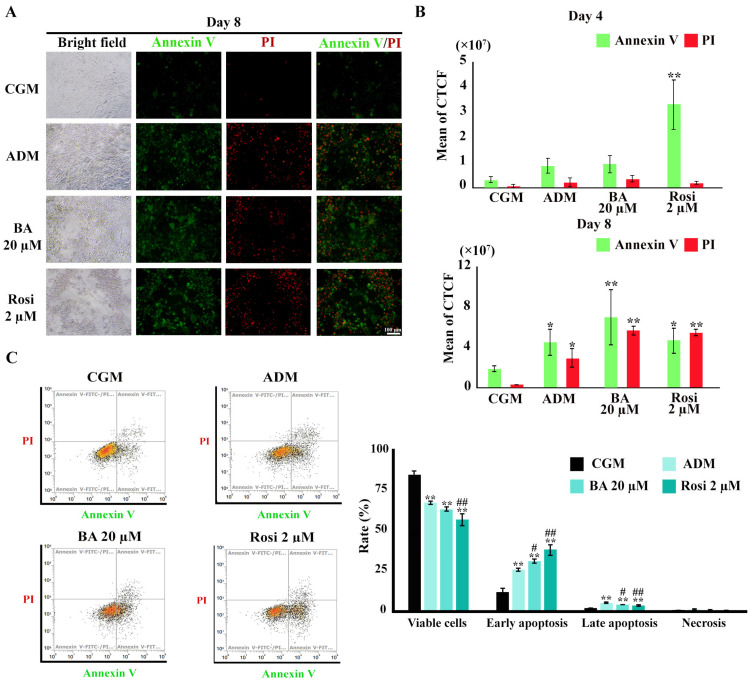
Annexin V/PI staining during adipogenesis of 3T3-L1. (**A**) Visualization of apoptotic cell death (×4 objective). (**B**) Semi-quantitative results from image analysis highlight increases in apoptotic cells by rosiglitazone on day 4 and ADM on day 8. (**C**) Flow cytometry results on day 8. Data are presented as mean ± SD. * *p* < 0.05 and ** *p* < 0.01 compared with CGM, while # *p* < 0.05 and ## *p* < 0.01 compared with ADM.

To investigate the induction of apoptosis during adipogenesis in 3T3-L1 cells, Annexin V/PI staining was utilized. Figure 9A illustrates the initial evidence of apoptotic cell death. Subsequent image analysis revealed a significant increase in Annexin V-stained cells following treatment with rosiglitazone by day 4 (Figure 9B). By day 8, an augmentation in late apoptosis, as evidenced by an expanded overlap in the Annexin V/PI stained area, was notably pronounced under adipogenic differentiation media (Figure 9B). However, an increase in Annexin or PI-stained areas could also separately reflect early apoptosis or necrosis. Therefore, flow cytometry analysis was performed to confirm these results.

Flow cytometry revealed a reduction in the viable cell ratio by 17% under ADM treatment (Figure 9C). The addition of rosiglitazone further decreased the viable cell ratio to 27.7% (Figure 9C). An increase in the ratio of both early and late apoptosis was observed with ADM treatment, which was further amplified by the addition of both betulinic acid and rosiglitazone (Figure 9C). However, necrosis cell death was minimal, ranging from about 0.2 to 0.6%, with no significant differences between treatment groups (Figure 9C). These findings delineate a clear enhancement of apoptotic cell death during adipogenesis, modulated by treatment with rosiglitazone and, to a certain extent, betulinic acid.

**Figure 10:**
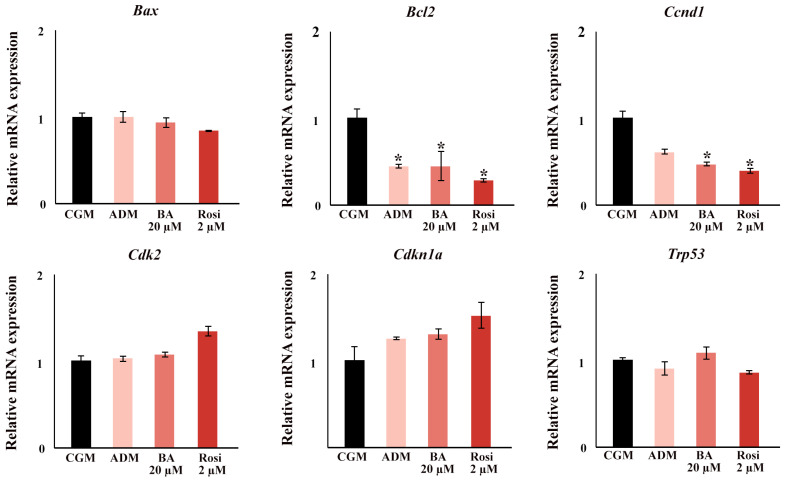
The relative mRNA expression of cell cycle and apoptosis gene. An asterisk (*) indicates *p* < 0.05 compared with CGM with a 2-fold cut-off.

In the gene expression analysis focusing on cell cycle and apoptotic factors, significant findings were observed during the early stages of adipogenesis. By day 2, ADM significantly induced a downregulation in the expression of B cell leukemia/lymphoma 2 (*Bcl2*), and supplementation with either rosiglitazone or betulinic acid led to a decrease in Cyclin D1 (*Ccnd1*) (Figure 10). Notably, no changes were detected in the expression of transformation-related protein 53 (*Trp53*), Cyclin-dependent kinase inhibitor 1A (*Cdkn1a*), Cyclin-dependent kinase 2 (*Cdk2*), and BCL2-associated X protein (*Bax*) at this time point (Figure 10).

## 4. Discussion

The 3T3-L1 cells used in this experiment are widely utilized as models for studying adipogenesis and assessing the anti-obesity potential of new extracts or drugs. Gaining a comprehensive understanding of their mechanisms is essential for the precise interpretation of experimental results. For the first time, this study reveals that apoptotic cell death occurs simultaneously during the adipogenesis of 3T3-L1 cells. Remarkably, we found that both the PPARG agonist, rosiglitazone, and the PPARG antagonist, betulinic acid, despite having differing effects on 3T3-L1 adipogenesis, can amplify this apoptotic cell death.

PPARG activation has been shown to induce apoptosis in various cancer types, including esophageal cancer, non-small cell lung carcinoma, and gastric cancer. In esophageal cancer cells, PPARG activation by rosiglitazone led to suppressed proliferation and induced apoptosis through the inhibition of the Toll-like receptor 4 (TLR4)-dependent MAPK pathway [43]. In this study, we found that rosiglitazone alone did not induce cell death in 3T3-L1 cells (Figure 6B). However, when combined with ADM, it significantly intensified apoptotic cell death.

Acting as a PPARG antagonist, betulinic acid has been reported to induce apoptosis in various cancer cell types through the activation of ROS production [44]. It also inhibited proliferation and migration against a wide range of cancers, including neuroblastoma, glioma, and breast cancer, as well as leukemia and multiple myeloma [45]. In non-cancer cells, betulinic acid was found to alter cell metabolic activity, resulting in increased glucose uptake without inducing cell death [46]. Consistent with these findings, our study observed that under growth media conditions, betulinic acid did not induce cell death. However, when combined with ADM, betulinic acid significantly enhanced apoptotic cell death.

Both betulinic acid and rosiglitazone, as modulators of PPARG, directly influence the process of adipogenesis [14,19,47,48,49]. In this study, similar results were established with betulinic acid reducing lipid droplet formation and rosiglitazone inducing large lipid droplet formation. However, an unexpected finding was that betulinic acid, despite being a PPARG antagonist, significantly upregulated the expression of *Fabp4*, a downstream target of PPARG, in 3T3-L1 cells. In contrast, the adipogenic induction media, which directly increased *Pparg2* expression (Figure 3B and Table S1), did not produce a corresponding increase in *Fabp4* expression. This discrepancy may be partially explained by the intrinsically high basal expression of *Fabp4* and *Pparg2* in 3T3-L1 cells, as indicated by their relatively low delta Ct values in Table S1, compared with other adipogenic markers such as *Adipoq* and *Cfd*. This suggests that the regulation of *Fabp4* by betulinic acid may involve alternative pathways beyond simple PPARG antagonism, reflecting a more complex regulatory network. Moreover, as an anti-inflammatory agent, betulinic acid has been shown to inhibit IL-1β-induced inflammation in human osteoarthritis chondrocytes, an effect that can be reversed by GW9662, a potent synthetic PPARG antagonist [50]. This functional contrast indicates that betulinic acid may engage additional, yet unidentified, signaling pathways beyond PPARG antagonism, potentially involving anti-inflammatory or oxidative stress-related mechanisms that could indirectly modulate *Fabp4* expression. This unique functional profile highlights the need for further studies to fully elucidate the diverse roles of betulinic acid in adipogenesis, inflammation, and metabolic regulation.

In addition to the unexpected *Fabp4* upregulation observed with betulinic acid, both betulinic acid and rosiglitazone significantly influenced the expression of other key adipogenic markers. For example, rosiglitazone strongly induced the expression of *Cebpa*, both with and without ADM, suggesting a central role in promoting lipid droplet formation and controlling stages of cell growth and death. Previous research also showed that *Cebpa* is essential for maintaining postmitotic growth arrest in adipocytes [51] and that overexpression of *Cebpa* alone, even without exogenous hormonal agents, was sufficient to initiate the adipocyte differentiation program in 3T3-L1 cells [52]. Additionally, rosiglitazone and betulinic acid enhanced the expression of *Adipoq* and *Cfd* during ADM supplementation. These adipogenic markers were also found to play critical roles in cell proliferation, cell cycle arrest, and apoptosis induction in other cells [53,54].

Recent reports have suggested that the antiapoptotic activity of adiponectin might contribute to its therapeutic potential during ischemia/reperfusion injury, although some studies have demonstrated that adiponectin induces apoptosis both in vitro and in vivo [55]. Moreover, adiponectin-induced anti-angiogenesis and antitumor activities involve caspase-mediated endothelial cell apoptosis [56], highlighting the complexity of its biological functions. Selective suppression of endothelial cell apoptosis by the high molecular weight form of adiponectin further underscores the specific actions of different adiponectin isoforms [57]. Additionally, injected adipsin has been shown to prevent beta-cell dedifferentiation and cell death in diabetic mice models [58]. However, it also promoted breast cancer growth and clonogenicity, indicating a dual role in both protecting against cell death and promoting tumor growth [59]. Similarly, overexpression of *Cebpa* not only induces apoptosis but also suppresses the proliferation and differentiation of myeloid cells, which could have significant implications for therapeutic strategies targeting these pathways [60]. While our current focus is primarily on transcriptional regulation, we acknowledge that future studies integrating protein expression analysis, such as Western blotting for markers such as Bcl2 and Ccnd1, could provide a more comprehensive understanding of the mechanistic pathways involved. This approach would help to clarify the post-transcriptional and post-translational modifications that may contribute to the observed apoptotic effects, offering deeper insights into the regulatory networks governing adipogenesis and apoptosis in 3T3-L1 cells.

The findings of this study also raise the possibility of future therapeutic applications. Given the ability of BA to modulate both adipogenic differentiation and apoptotic cell death, it may hold potential as a dual-action therapeutic agent for metabolic disorders, including obesity and type II diabetes. The capacity to simultaneously influence lipid metabolism and programmed cell death could offer a novel strategy for managing these conditions, particularly if further studies confirm its efficacy in more complex in vivo models.

## 5. Conclusions

This study provides novel insights into the dual role of PPARG modulators, betulinic acid, and rosiglitazone, in regulating both adipogenic differentiation and apoptotic cell death in 3T3-L1 cells. While rosiglitazone enhanced lipid droplet formation and induced the early expression of key adipogenic markers such as *Cebpa* and *Adipoq*, betulinic acid exerted an inhibitory effect on lipid accumulation, particularly when administered continuously across the differentiation timeline. Importantly, both compounds significantly amplified apoptosis in the context of adipogenic induction, a phenomenon that escaped detection by conventional cell viability assays but was clearly demonstrated through LDH release, Annexin V/PI staining, and flow cytometry analysis.

The simultaneous occurrence of adipogenesis and apoptosis suggests a tightly regulated balance between differentiation and programmed cell death, likely mediated by PPARG-dependent and independent mechanisms. The findings also underscore the complexity of interpreting cell viability in metabolically dynamic systems and point to the necessity of using complementary assays to evaluate cytotoxicity in adipogenic studies. These results have broader implications for the use of PPARG-targeting agents in obesity and metabolic disease research and provide a foundation for future studies investigating the molecular interplay between differentiation, metabolism, and cell death. These results have broader implications for the use of PPARG-targeting agents in obesity and metabolic disease research and provide a foundation for future studies investigating the molecular interplay between differentiation, metabolism, and cell death, potentially informing the development of novel therapeutic approaches.

## Data Availability

The original contributions presented in this study are included in the article/Supplementary Materials. Further inquiries can be directed to the corresponding author.

## Supplementary Materials

The following supporting information can be downloaded at: https://www.mdpi.com/article/10.3390/biomedicines13061340/s1, Figure S1: 3T3-L1 morphology in complete growth media (CGM) (A), and in adipogenic media (ADM) (B) supplemented with 20 µM betulinic acid (BA) and 2 µM rosiglitazone; Table S1: ΔCt values of adipogenic genes in CGM and ADM groups on Day 2 and Day 6. Gene expression was normalized to the 18S rRNA housekeeping gene. Data are presented as mean ± standard deviation (SD) from n = 3 independent experiments.

## Abbreviations

The following abbreviations are used in this manuscript:

MCE	Mitotic clonal expansion
TZDs	Thiazolidinediones
PPARG	Peroxisome proliferator-activated receptor gamma
JCRB	Research Bioresources Cell Bank
CGM	Complete growth medium
ADM	Adipogenic differentiation media
IBMX	Isobutylmethylxanthine
PBS	Phosphate-buffered saline
LDH	Lactate dehydrogenase
PI	Propidium iodide
CTCF	The corrected total cell fluorescence
BA	Betulinic acid
Rosi	Rosiglitazone
*Adipoq*	Adiponectin
*Pparg2*	Peroxisome proliferator-activated receptor gamma 2
*Cfd*	Small molecule-induced complement factor D/Adipsin
*Fabp4*	Fatty acid binding protein 4
Bax	BCL2-associated X protein
Bcl2	B cell leukemia/lymphoma 2
Ccnd1	Cyclin D1
Cdk2	Cyclin-dependent kinase 2
Cdkn1a	Cyclin-dependent kinase inhibitor 1A
Trp53	Transformation-related protein 53
