# Supplementary material for: Dual Modulation of Adipogenesis and Apoptosis by PPARG Agonist Rosiglitazone and Antagonist Betulinic Acid in 3T3-L1 Cells
Source: Biomedicines. 2025 May 30;13(6):1340. doi: 10.3390/biomedicines13061340 (PMC12190099; doi:10.3390/biomedicines13061340)
Supplement: Supplementary file 1 [file biomedicines-13-01340-s001.zip › biomedicines-3643707-supplementary/Table S1.pdf]

Table S1. ΔCt values of adipogenic genes in CGM and ADM groups at Day 2 and Day 6. Gene expression was normalized to the 18S rRNA housekeeping gene. Data are presented as mean ± standard deviation (SD) from n = 3 independent experiments.

Day 2

	**ΔCt values**			
	Adipoq	***Cfd***	***Cebpα***	***Pparg2***
**CGM**	18.09 ± 0.03	20.00 ± 0.15	19.51 ± 0.11	15.37 ± 0.13
**ADM**	16.34 ± 0.05	19.32 ± 0.01	19.28 ± 0.07	14.22 ± 0.03

Day 6

	**ΔCt values**			
	Adipoq	***Cfd***	***Fabp4***	***Lep***
**CGM**	18.15 ± 0.06	19.48 ± 0.08	14.71 ± 0.05	19.16 ± 0.06
**ADM**	13.13 ± 0.15	13.21 ± 0.07	15.09 ± 0.03	19.49 ± 0.06
