# Supplementary figures and images for: Dual Modulation of Adipogenesis and Apoptosis by PPARG Agonist Rosiglitazone and Antagonist Betulinic Acid in 3T3-L1 Cells
Source: Biomedicines. 2025 May 30;13(6):1340. doi: 10.3390/biomedicines13061340 (PMC12190099; doi:10.3390/biomedicines13061340)

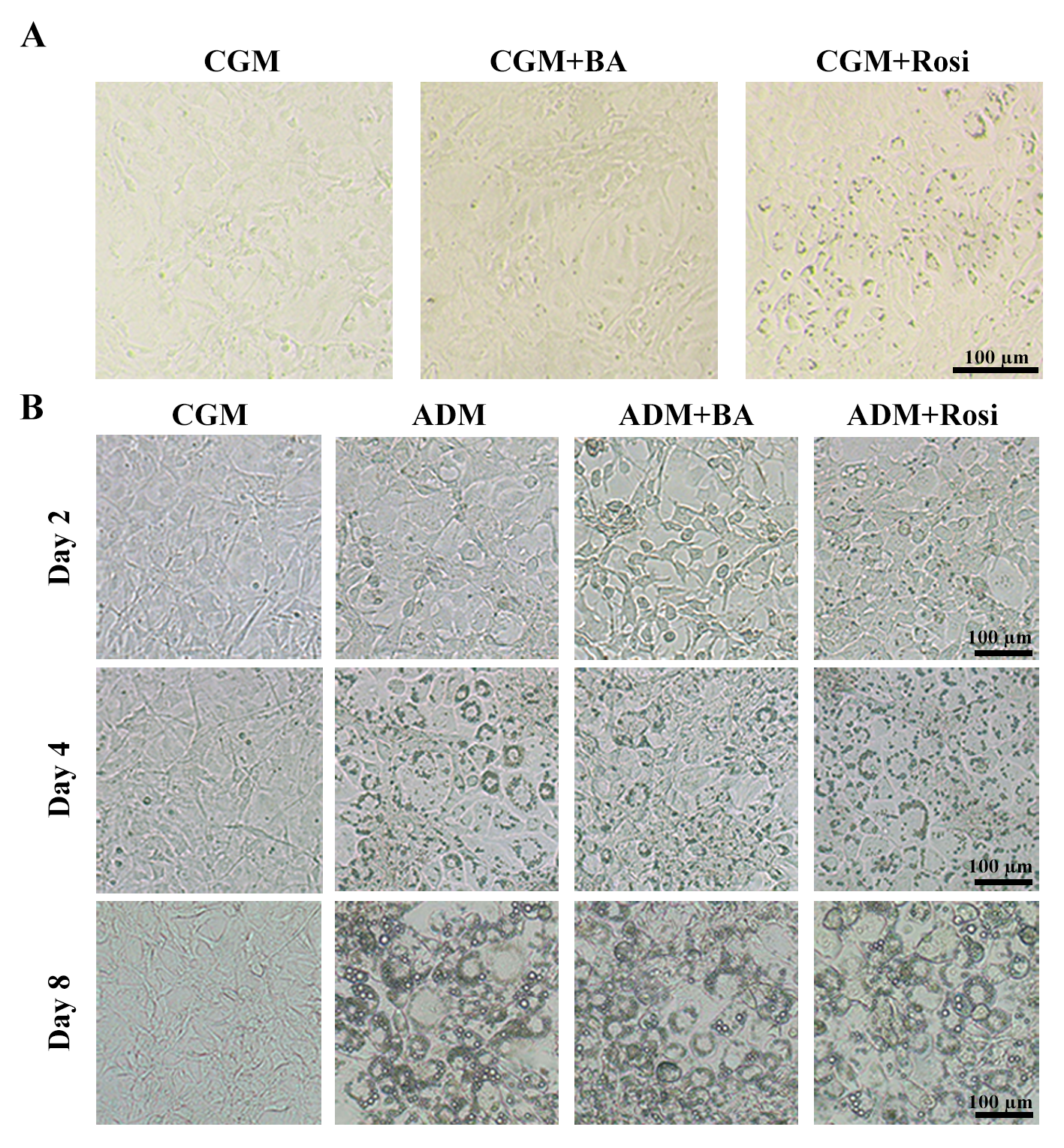

Supplement: Supplementary file 1 [file biomedicines-13-01340-s001.zip › biomedicines-3643707-supplementary/Figure S1.tif]
